# WormSORT: A detection-based multiple object tracking model for individual silkworms in breeding environments

**DOI:** 10.1371/journal.pcbi.1014410

**Published:** 2026-06-15

**Authors:** Hongkang Shi, Linbo Li, Shiping Zhu, Haibo He, Minghui Zhu, Jianfei Zhang

**Affiliations:** 1 Sericultural Research Institute, Sichuan Academy of Agricultural Sciences, Nanchong, Sichuan China; 2 College of Engineering and Technology, Southwest University, Beibei, Chongqing, China; Zhejiang University of Technology, CHINA

## Abstract

Variety breeding has long been a cornerstone of high-quality agriculture, and recent advances in artificial intelligence have opened new avenues for accelerating biological breeding. In this study, we applied multiple object tracking (MOT) technology to silkworm breeding to achieve efficient, non-invasive, and dynamic individual monitoring. Unlike pedestrian or vehicle tracking, silkworms pose unique challenges for MOT due to their small size, dense distribution, and high inter-individual similarity, which complicate accurate tracking and behavioral analysis. To address these issues, we propose WormSORT, an enhanced tracking method based on a tracking-by-detection framework with an optimized data association strategy. A pre-trained detection model identifies silkworms in each frame, and deep feature vectors are extracted using a re-identification network. Identity association is first performed using Intersection over Union (IoU) matching, followed by deep feature similarity for unmatched cases, improving both tracking accuracy and reliability. To further enhance tracking stability, we introduce a candidate input padding mechanism, including IoU padding and feature padding, ensuring that high-confidence unmatched trajectories and detections remain involved in the matching process. To validate the proposed tracking strategy, we constructed two multiple silkworm tracking (MST) datasets: MST-50, containing approximately 50 individuals over 1000 frames, and MST-100, containing approximately 100 individuals over 1200 frames. Experimental results demonstrate that WormSORT outperforms existing methods, including DeepSORT, StrongSORT, OCSORT, ByteTrack, and BotSORT, achieving superior tracking performance. This study provides a valuable reference for silkworm tracking and behavioral analysis, contributing to the advancement of high-quality silkworm rearing and management.

## Author summary

Artificial intelligence (AI) has achieved remarkable progress in computational biology, with notable success in tasks such as protein structure prediction, molecular property modeling, and drug discovery. In the field of silkworm breeding, AI also shows great potential to accelerate research and production processes, enabling large-scale counting, behavior recognition, and health condition assessment. This study focuses on leveraging deep learning techniques to extract crawling trajectories of silkworms through multiple object tracking. Accurate trajectory information provides essential support for analyzing individual movement patterns, modeling group interactions, and evaluating traits related to growth and health conditions. Therefore, it serves as a fundamental tool that bridges counting, behavior analysis, and health assessment tasks. However, most existing multiple object tracking methods are designed for pedestrians or vehicles. In contrast, silkworm tracking presents unique challenges, including dense distributions, highly non-linear motion patterns, and strong visual similarity among individuals, which limit the direct applicability of existing approaches. To address these challenges, this work proposes a multiple object tracking method tailored for silkworm scenarios and constructs a dedicated silkworm tracking dataset to validate its effectiveness.

## 1. Introduction

Originating over 5,000 years ago in ancient China, the silkworm is a key economic insect primarily cultivated for silk production. It played a pivotal role in establishing the Silk Road and continues to be widely farmed in regions such as China, India, and Southeast Asia, remaining an integral part of both agriculture and the textile industry [1, 2]. Silkworm farming entails various visual tasks, including counting individuals for breeding or conserving genetic resources, determining whether silkworms are dormant or feeding for precise rearing, and detecting signs of disease. Historically, these tasks were performed manually, which is labor-intensive, requires substantial expertise, and lacks dynamic recognition capabilities.

In recent years, advances in artificial intelligence (AI), particularly deep learning applied to biological data, have increasingly been used in computational biology to analyze complex biological datasets. A landmark achievement in this area is the AI-driven prediction of protein structures, exemplified by the AlphaFold model developed by Abramson et al. [3]. More recently, Guo et al. [4] applied generative AI to design protein switches functional within live bacterial cells, which can be connected to electrodes to generate electrical signals, providing new insights for biotechnology, medicine, and biosensing. Zhao et al. [5] proposed a universal deconvolution framework for estimating cell-type abundances from transcriptomic, proteomic, and metabolomic data. Within this context, silkworm research has also benefited from AI-driven methods, including pupal sex identification [6], individual counting [7], and molting-awakening rate [8].

Multiple object tracking (MOT), a key subfield of visual deep learning, involves identifying and assigning an identity to each object in the first frame of a video and subsequently locating and associating the same objects across frames to trace their trajectories [9 - 11]. Tracked objects typically include pedestrians, vehicles, and other organisms. In biological research, MOT has considerable potential for automated individual tracking and behavior analysis, enabling high-throughput and non-invasive monitoring of organisms, which is essential for understanding developmental, behavioral, and phenotypic dynamics. Compared with pose estimation or semantic segmentation, MOT requires only bounding box annotations, enabling reuse of existing detection datasets and reducing labeling effort; it also naturally generates individual trajectories for behavior analysis, is computationally efficient, and more robust to occlusions and high inter-individual similarity. By leveraging MOT, individual silkworms can be dynamically identified, providing an efficient, non-invasive method for counting and enabling tracking of crawling trajectories for behavioral analysis and disease detection.

However, existing MOT methods have been primarily developed for pedestrians or vehicles, which typically move linearly at roughly constant speeds and possess distinctive visual features. In contrast, silkworms are small, densely distributed, and visually similar, exhibiting irregular and non-linear movements. These characteristics render conventional tracking approaches inadequate, highlighting the need for a MOT model specifically tailored to silkworms.

In this study, we propose WormSORT, a MOT method designed specifically for silkworms. The method leverages the relatively stable positions of silkworms. Identity association is first performed using Intersection over Union (IoU) matching, followed by deep feature matching to improve efficiency. To enhance tracking stability, IoU and feature padding are introduced to ensure high-confidence trajectories and detections remain in the matching process. Experiments on MST-50 (approximately 50 individuals over 1000 frames) and MST-100 (approximately 100 individuals over 1200 frames) demonstrate that WormSORT achieves superior tracking performance. The main contributions of this paper are as follows:

(1) The application of artificial intelligence to traditional silkworm farming, introducing a visual deep learning-based MOT method for silkworms.
(2) The introduction of WormSORT, a silkworm-specific MOT model that outperforms baseline methods in tracking accuracy.
(3) The proposal of a dynamic individual counting and trajectory acquisition method, providing tools for behavioral analysis and disease identification.

The structure of this study is as follows: Section 2 reviews related works, Section 3 details the materials and methods, including the silkworm MOT dataset and the proposed WormSORT structure, Section 4 presents the experimental results, Section 5 provides a discussion, and Section 6 concludes the study.

## 2. Related works

### 2.1. Deep learning in biology research

Thanks to its significant advantages in data-driven inference, deep learning has been widely applied in biological research, greatly accelerating the progress of modern biology. At the molecular and genomic level, DeWeirdt et al. [12] combined language models and machine learning algorithms to develop a predictive model of prokaryotic immune systems, capable of accurate predictions on previously unseen data. Charlier et al. [13] compared different neural network architectures for CRISPR-Cas9 off-target prediction and proposed a similarity-based transfer learning approach. Afting et al. [14] utilize deep learning to predict the differentiation path and resulting tissues in retinal organoids well before they become visually discernible. Zhang et al. [15] developed a framework to infer cell line-specific molecular features from transcriptional regulation networks, integrating three modalities of input data, widely used in genome annotation. At the organismal level, deep learning has been applied to behavior tracking and biomechanics. For example, Ulrich et al. [16] employed pose estimation to track movements of worker bees and queens, combining machine learning to extract behavioral metrics and establish a bee behavior tracking system. Melis et al. [17] investigated insect wing biomechanics, using convolutional neural networks to predict the relationship between controlling muscle activity and wing motion. Han et al. [18] collected video recordings of two mice, applied multi-stage artificial neural networks to identify individuals and their postures, and generated three-dimensional trajectories for unsupervised multi-animal behavior recognition.

In silkworm research, deep learning has been applied to various tasks. Guo et al. [6] developed a posture correction algorithm for pupae, improving sex identification accuracy. He et al. [19] combined near-infrared spectroscopy with deep learning to identify developmental morphology, sex, and variety. Shi et al. [20] proposed an enhanced object detection method for individual recognition. These studies illustrate the applicability of deep learning in silkworm research, providing a reference for MOT and related downstream tasks.

### 2.2. Current studies in MOT

Multiple object tracking (MOT) is a significant subfield of visual deep learning with applications in autonomous driving, robotics, and smart agriculture. MOT methods are typically classified into Tracking by Detection (TBD) and End-to-End approaches [11]. TBD methods, which offer significant advantages in tracking accuracy, have gained considerable attention in the industry. SORT and DeepSORT [10] are pioneering methods in this field, using Kalman filters to update trajectory states and the Hungarian algorithm to associate deep feature vectors between trackers and detected objects, resulting in substantial improvements in tracking performance. Li et al. [21] proposed a real-time detection and counting method for wheat ears based on DeepSORT. Zhang et al. [22] integrated convolutional auto-encoders into DeepSORT to enhance appearance feature extraction, introducing a tracking method for young honey peach fruits. Cao et al. [23] developed a dynamic counting method for sheep using DeepSORT. Meng et al. [24] proposed a real-time tracking method for cherry tomato count, utilizing a lightweight network to extract deep features and optimize matching efficiency. Tu et al. [25] introduced a passion fruit yield prediction method based on OC-SORT and YOLOv8. Optimizing data association methods remains a central focus in MOT research. Li et al. [26] described a multi-scale fusion network for object feature matching, employing similarity matrix loss to calculate associations between objects in different frames. Zhang et al. [27] used a spatiotemporal association model to extract historical trajectory correlations and employed graph neural networks to fuse appearance and motion information. Wang et al. [28] proposed a joint detection and matching method utilizing neighboring frame position prediction and inter-frame relationships to predict object positions in the next frame. Liang et al. [29] introduced a tracking algorithm for aerial vehicles, relying on a Gaussian distance module and an observation-centered Kalman filter. Muzaddid and Beksi [30] proposed a tracking method based on linear relationships between adjacent trajectory positions, using dense optical flow and particle filtering to guide each tracker.

These studies highlight the broad applications of MOT and provide valuable references for silkworm tracking. Building upon this prior work, we optimize data matching strategies and propose a MOT method specifically tailored to silkworms.

## 3. Materials and methods

### 3.1. Silkworm MOT dataset

In this study, silkworm videos were collected under real-world conditions, preprocessed, and used to construct two MOT datasets. Data acquisition was conducted on September 10, 2023, at the Sericultural Research Institute of the Sichuan Academy of Agricultural Sciences, Sichuan Province, China. A mobile camera (Moker High-Speed USB 3.0; resolution: 2592 × 1944; frame rate: 25 FPS) was mounted vertically above the rearing boxes to capture video data. The environment was illuminated by incandescent lamps, with the temperature maintained at 29 °C and relative humidity at 80%.

In large-scale silkworm farming, high-density rearing often results in frequent occlusions caused by overlapping mulberry leaves or individual blockage, posing significant challenges for data collection and annotation. Given that silkworm MOT is intended for biological applications such as strain breeding, behavior analysis, and disease prediction, this study focused on a breeding environment with relatively low population density. After a period of feeding, silkworms typically emerge from beneath the mulberry leaves and consume most of them, thereby substantially reducing occlusion.

**Fig 1:**
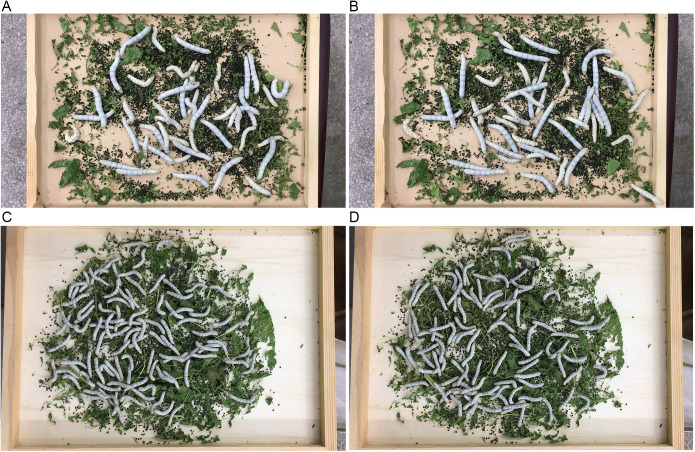
Schematic images of silkworm MOT dataset. **(a)** Frame 1 of MST-50. **(b)** Frame 300 of MST-50. **(c)** Frame 1 of MST-100 **(d)** Frame 500 of MST-100.

Two videos were recorded in this breeding environment, each with a duration of approximately 1.5 hours. Due to the slow crawling speed of silkworms, frames were sampled at 3-second intervals, resulting in two datasets: MST-50 (multi-silkworm tracking with approximately 50 individuals across 1,000 frames) and MST-100 (approximately 100 individuals across 1,200 frames). The spatial distribution of silkworms in the dataset is illustrated in Fig 1. Following the MOT17 dataset construction methodology [31], the position of each silkworm was manually annotated across all frames using LabelImg, yielding over 50,000 and 120,000 individual annotations for MST-50 and MST-100, respectively. These annotations were extracted from the generated XML files and converted into text format to form the final MOT dataset.

Table 1 presents a sample of the annotation file, where each line corresponds to a silkworm instance in a single frame, including the frame index, identity ID, bounding box center coordinates, and bounding box dimensions.

**Table 1:** Schematic of the annotation file.

Frame Number	Individual ID	X Coordinate	Y Coordinate	Width	Height
1	1	242	195	81	83
2	1	242	207	73	70
3	1	234	215	73	70
4	1	234	215	73	70
5	1	233	214	73	70
6	1	233	215	73	70

### 3.2. WormSORT for silkworm MOT

**Fig 2:**
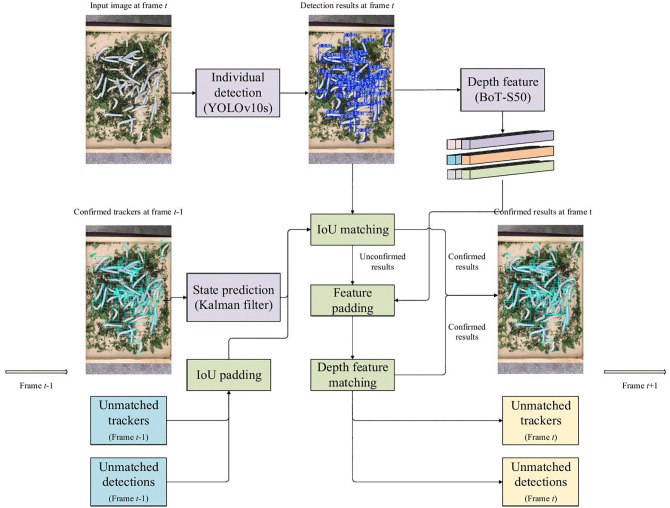
Structure of WormSORT.

To enable precise tracking of individual silkworms in a breeding environment, we propose a multiple object tracking (MOT) method based on a tracking-by-detection (TBD) framework, termed WormSORT. As illustrated in Fig 2, WormSORT consists of three main components: a detector (the pretrained YOLOv10s), a feature extraction model (BoT-S50 pretrained on the DukeMTMC-ReID dataset), and a data association module. The detector is employed to localize silkworms in each frame, while BoT-S50 extracts appearance features of the detected targets. Data association is first performed using the Intersection over Union (IoU) metric, followed by feature-based matching to improve robustness and accuracy. To further enhance the matching performance, we introduce IoU padding and feature padding strategies, which allow high-confidence unmatched trackers and detections to remain in the association process.

#### 3.2.1. Algorithm flow of WormSORT.

When WormSORT is used to track silkworm trajectories across consecutive frames, YOLOv10s [32] first localizes the silkworms, and the pre-trained BoT-S50 [33] extracts their depth features. A detection set is generated: $D_{\text{1}}=\left\{d_{\text{1}},d_{\text{2}},...,d_{n}\right\}$, where each detection$d_{i}$ contains position information, a depth feature vector, and a detection confidence score. Since the initial tracking set $T_{\text{0}}$ is empty, no association is performed in the first frame. Instead, the detection set is directly initialized as the tracking set: $T_{\text{1}}=\left\{t_{\text{1}},t_{\text{2}},...,t_{n}\right\}$, where each tracked individuals $t_{i}$ is assigned an identity ID, position, state vector$x_{t}$, and covariance matrix $P_{t}$, which are subsequently updated using a Kalman filter In addition, each track maintains its depth feature vectors and the number of confirmed frames.

In the second frame, the same detection and feature extraction process is repeated, producing a new detection set $D_{\text{2}}=\left\{d_{\text{1}},d_{\text{2}},...,d_{m}\right\}$. Since the trajectories in $T_{\text{1}}$ have not yet been confirmed, detections in $D_{\text{2}}$ are initialized as new trajectories and merged with $T_{\text{1}}$ to form $T_{\text{2}}$. The Kalman filter is then applied to predict the likely positions of each trajectory in the next frame.

From the third frame onward, detections $D_{t}$ and predicted positions ${\hat{x}}_{t|t-\text{1}}$ $\left\{d_{i}\in D_{t}\right\}$ are used to perform association. First, the IoU values between $\left\{d_{i}\in D_{t}\right\}$ and $\left\{{\hat{x}}_{t|t-1}\in T_{t-1}\right\}$ are calculated to form a cost matrix. The Hungarian algorithm is applied to obtain the first-round associations. Unmatched detections are further compared to existing tracks using depth features. The cosine distance between detection and track feature vectors forms a new cost matrix, which is again solved using the Hungarian algorithm.

Unmatched detections are initialized a s new tracks and added to the tracking set. Unmatched tracks are marked as tentative; if a tentative track remains unconfirmed for more than a predefined number of frames $\tau_{tentative}$, it is removed. This iterative process yields the complete crawling trajectories of silkworms. A detailed stepwise flow of WormSORT is provided in Table 2.

**Table 2:** Algorithmic flow of WormSORT.

Step	Description
1. Initial detection set	Silkworms are detected using YOLOv10s and BoT-S50, generating a detection set: $D_{t}=\left\{d_{\text{1}},d_{\text{2}},...,d_{n}\right\}$, where each detection$d_{i}$ contains position information and depth features $\Phi_{i}$.
2. Initial tracking set	The initial detection set is considered as the tracking set: $T_{t}=\left\{t_{\text{1}},t_{\text{2}},...,t_{m}\right\}$, where each tracked individual $t_{i}$ contains position, speed, state, and other attributes.
3. Prediction update	The Kalman filter is used to predict the state of the tracking set for the next frame, yielding predicted positions: ${\hat{x}}_{t}$.
4. IoU Matching	Matching is performed by calculating the IoU between$D_{t}$ and $T_{t}$:$IoU\text{(}d_{i},t_{j}\text{)}\geq \tau_{IoU}$.
5. Depth Feature Matching	For unmatched detections $d_{u}\in D_{u}$ and unmatched trackers $t_{u}\in T_{u}$, the cosine distance between their depth features is calculated:$cos\text{(}\Phi_{d},\Phi_{t}\text{)=}\frac{\Phi_{d}*\Phi_{t}}{\|\Phi_{d}\|\|\Phi_{t}\|}\leq \tau_{cos}$.
6. State update	Matched trackers: update trajectory state (position, depth features, etc.). Unmatched trackers: mark as “unconfirmed” and increment the number of frames without updates. Unmatched detections: initialize as a new trackers and add them to $T_{t}$。
7. Final Output	Return the updated tracking set $T_{t}$, including: (1) the most recent positions and motion states of all confirmed trajectories; (2) depth-feature matching information.

#### 3.2.2. Data association of WormSORT.

The data association procedure in WormSORT is conceptually similar to DeepSORT, which relies on IoU and feature-based matching. However, unlike DeepSORT, WormSORT first performs IoU-based matching, followed by feature-based matching. This design leverages the observation that silkworm positions and states exhibit temporal continuity, thereby reducing the likelihood of mismatches.

The IoU between a detection and a predicted track is defined as:


$$\begin{array}{c} IoU\text{(}\overset{t}{\underset{i}{d}},\overset{t}{\underset{j}{\hat{x}}}\text{)}=\frac{\left|\overset{t}{\underset{i}{d}}\cap \overset{t}{\underset{j}{\hat{x}}}\right|}{\left|\overset{t}{\underset{i}{d}}\cup \overset{t}{\underset{j}{\hat{x}}}\right|} \end{array}$$ (1)

The corresponding cost matrix is expressed as:


$$\begin{array}{c} C_{IoU}\text{[}i,j\text{]}=1-IoU\text{(}\overset{t}{\underset{j}{d}}\text{,}\overset{t}{\underset{j}{\hat{x}}}\text{)} \end{array}$$ (2)

where $\overset{t}{\underset{j}{d}}$ represents the position of the i-th detection in the frame t, and $\overset{t}{\underset{j}{\hat{x}}}$ denotes the predicted position of the j-th tracker after Kalman filtering.

The Hungarian algorithm was used to explore the optimal matching solution by solving the following optimization problem:


$$\begin{array}{c} {ℳ}_{IoU}={argmin}_{ℳ}\sum_{\text{(}i,j\text{)}\in ℳ}C_{IoU}\text{[}i\text{,}j\text{]} \end{array}$$ (3)

where ℳ represents the matching set, $D_{i}$ is the detection set, and $T_{i}$ is the tracker set.

For unmatched detections and trackers, the cosine distance between their depth feature vectors is calculated to form the cost matrix:


$$\begin{array}{c} C_{feature}\text{[}i\text{,}j\text{]}=1-\frac{f_{i}*f_{j}}{\|f_{i}\|\|f_{j}\|} \end{array}$$ (4)

where $f_{i}$ denotes the depth feature vector of the i-th detection, and$f_{j}$ denotes the depth features vector of the j-th tracker.

The Hungarian algorithm is then applied to minimize the feature-based cost matrix:


$$\begin{array}{c} {ℳ}_{Feature}={argmin}_{ℳ}\sum_{\text{(}i,j\text{)}\in ℳ}C_{Feature}\text{[}i\text{,}j\text{]} \end{array}$$ (5)

The final matching result is obtained by combining both stages:


$$\begin{array}{c} ℳ={ℳ}_{Feature}\cup {ℳ}_{IoU} \end{array}$$ (6)

Thus, the data association strategy in WormSORT integrates the efficiency of IoU-based matching with the accuracy of depth feature matching, thereby ensuring both robustness and tracking efficiency, which is particularly effective given the relatively stable positions of silkworms.

#### 3.2.3. Candidate input padding mechanism.

Multiple object tracking methods based on object detection rely heavily on the accuracy of the detector. However, in silkworm tracking, the close proximity of individuals makes the system highly sensitive to minor detection failures, which can severely reduce the accuracy of data association. Moreover, the high visual similarity among silkworms often causes overlapping bounding boxes, diminishing the discriminative power of depth features.

To address these challenges, WormSORT introduces two complementary padding strategies: IoU-padding, which exploits the spatial continuity of trajectories, and feature-padding, which leverages the similarity of depth features. Together, these mechanisms compensate for missed detections and reduce mismatches by augmenting the candidate set before association.

(1) IoU padding

Prior to IoU-based matching, the mechanism considers not only the trajectories confirmed in the previous frame but also unconfirmed trajectories and detections carried over from earlier frames. Specifically, if the geometric position $c(\Delta \overset{t-1}{\underset{k}{T}})$ of an unmatched tracker in the previous frame and the center point $c\left(\overset{t-10}{\underset{j}{D}}\right)$ of a detection box within the following ten frames satisfy the Euclidean distance condition, the trajectory is retained for further consideration:


$$\begin{array}{c} d(\Delta \overset{t-1}{\underset{k}{T}},D_{j})={\|c(\Delta \overset{t-1}{\underset{k}{T}})-c\left(\overset{t-\text{10}}{\underset{j}{D}}\right)\|}_{\text{2}}\leq \delta \end{array}$$ (7)

where δ denotes the predefined motion-range threshold, set to 5. $\Delta \overset{t-1}{\underset{k}{T}}$ represents the set of unmatched trackers from the previous frame.

Through this augmentation, unconfirmed yet high-quality trackers from the past ten frames are reintroduced into the matching set. Such trackers may have been excluded because two silkworms were positioned in close proximity, leading them to appear as secondary costs in the assignment matrix, or due to missed detections that prevented their confirmation in earlier frames. Accordingly, the candidate tracker set is defined as:


$$\begin{array}{c} Candidates_{IoU}=\left\{(\Delta \overset{t-1}{\underset{k}{T}}|d(\Delta \overset{t-1}{\underset{k}{T}},D_{j})\leq \delta )\cup \overset{t-1}{\underset{k}{\text{T}}}\right\} \end{array}$$ (8)

where $\overset{t-1}{\underset{k}{T}}$ denotes the set of confirmed trajectories from the previous frame.

This strategy constitutes the IoU padding mechanism, ensuring that spatially consistent yet unconfirmed trackers remain available for matching.

(2) Feature padding

During the deep feature matching stage, supplementary matching is performed only for detection boxes that failed in IoU-based matching. In addition, trajectories in $\Delta \overset{t-1}{\underset{k}{T}}$ whose cosine similarities to certain detection boxes within the most recent ten frames satisfy the following condition are also included:


$$\begin{array}{c} e\text{(}\Delta \overset{t-1}{\underset{k}{T}},D_{j}\text{)}=\frac{f\text{(}\Delta \overset{t-1}{\underset{k}{T}}\text{)}*f\text{(}D_{j}\text{)}}{\|f\text{(}\Delta \overset{t-1}{\underset{k}{T}}\text{)}\|\|f\text{(}D_{j}\text{)}\|}\geq \varepsilon \end{array}$$ (9)

where $\Delta T_{IoU}$ denotes the set of trajectories unconfirmed by IoU matching, and ε denotes the predefined cosine similarity threshold, set to 0.8.

Unmatched trajectories that satisfy this condition are incorporated into the feature candidate set:


$$\begin{array}{c} Candidates_{F}=\left\{\Delta T_{IoU}\cup (\Delta \overset{t-1}{\underset{k}{T}}|e(\Delta \overset{t-1}{\underset{k}{T}},D_{j})\geq \varepsilon ))\right\} \end{array}$$ (10)

This strategy constitutes the feature padding mechanism, ensuring that high-confidence trajectories based on feature similarity are preserved.

Taken together, WormSORT employs both IoU-padding and feature-padding mechanisms, which jointly compensate for detection failures and mismatches. By fully exploiting both spatial continuity and feature similarity, these mechanisms enhance robustness and ensure continuity in silkworm trajectory tracking.

#### 3.2.4. Detector training and appearance feature extraction.

WormSORT utilizes YOLOv10s for its favorable balance between efficiency and detection accuracy. Architecturally, it follows the standard YOLO paradigm, consisting of a backbone, a neck, and three detection heads, and performs object localization and classification via fully convolutional operations. YOLOv10 further introduces a consistent dual-assignment strategy for NMS-free training to mitigate redundant predictions. Additionally, a lightweight classification head, spatial–channel decoupled downsampling, and rank-guided blocks are employed to reduce computational redundancy. The integration of large-kernel convolutions and an efficient partial self-attention module further enhances representational capacity with minimal overhead.

**Fig 3:**
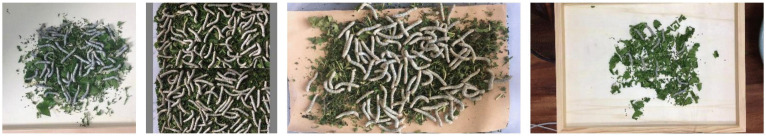
Image examples of object detection dataset.

To adapt the detector to the silkworm tracking scenario, YOLOv10s was trained on a dedicated dataset comprising 1,526 images with 83,698 annotated instances, averaging over 50 silkworms per image. Representative training samples are shown in Fig 3, demonstrating significant variations in object scale and spatial distribution. The dataset was split into training and validation sets at a ratio of 7: 3. During training, Mosaic augmentation was applied, with an input resolution of 720 × 720 pixels. Optimization was performed using the Adam optimizer with the CIoU loss for 300 epochs, an initial learning rate of 0.001, and a cosine decay schedule.

The trained detector achieved a recall of 97.37%, a precision of 97.84%, and an mAP@50 of 98.63% on the MST-50 dataset. Despite these competitive detection results, the tracking performance remained suboptimal when directly applied to MOT. To address this issue, 500 additional images were sampled from MST-50 at regular intervals and incorporated into the training set for further fine-tuning. The refined detector achieved a recall of 99.79%, a precision of 99.81%, and an mAP@50 of 99.50%, and was subsequently used for experiments on MST-50. The impact of the number of sampled images on tracking performance in MST-100 is further analyzed in Section 4.2.

For appearance modeling, a BoT-S50 model with a ResNet-50 backbone, implemented in the FastReID framework and pretrained on the DukeMTMC-ReID dataset, is used to extract discriminative appearance features, which aid in robust data association during tracking. Based on the detector’s predictions, the position of each silkworm is determined, and its bounding box is extracted. The image is then cropped, resized to 256 × 128 pixels, and passed through the model to extract a 1024-dimensional feature vector, which serves as the appearance feature.

### 3.3. Experimental setting and evaluation metrics

Experiments were conducted on a DELL 5820 workstation equipped with an Intel Core i7-9800X CPU and NVIDIA RTX 2080Ti GPUs (11 GB memory), using CUDA 10.0. The operating system was Windows 10 Professional (64-bit). All models were implemented in Python 3.7 using the PyTorch 1.7 framework, with auxiliary libraries including NumPy, Keras, and OpenCV.

To evaluate the impact of the detector on tracking performance on the MST-100 dataset (Section 4.2), two training strategies were compared: training from scratch and transfer learning. Additional training images were uniformly sampled from MST-100 and incrementally incorporated into the original training set. For training from scratch, the same hyperparameters as those used for the MST-50 detector were adopted. For transfer learning, training was initialized with 100 epochs, and the number of epochs was increased by 10 with each increment of added training images. In each transfer learning stage, training was initialized from the baseline detector weights, without sequential fine-tuning across stages. After training, the detector was evaluated on MST using recall, precision, F1-score, and mAP@50.

For MOT evaluation, the detection confidence threshold was set to 0.3, the cosine similarity threshold for feature matching to 0.4, and the intersection-over-union (IoU) threshold to 0.7. The maximum retention length of unconfirmed trajectories was set to 30 frames.

Tracking performance was evaluated using five widely adopted metrics: Multi-Object Tracking Accuracy (MOTA), IDF1 score (IDF1), Association Accuracy (AssA), Detection Accuracy (DetA), and Higher-Order Tracking Accuracy (HOTA). Their formulations and corresponding definitions are as follows.:


$$\begin{array}{c} \text{MOTA}=1-\frac{\sum_{t}\left({\text{FN}}_{\text{t}}{\text{+FP}}_{\text{t}}{\text{+IDSW}}_{\text{t}}\right)}{\sum_{t}{\text{GT}}_{\text{t}}} \end{array}$$ (11)

where ${\text{GT}}_{\text{t}}$ denotes the number of ground-truth objects at time t, ${\text{FN}}_{\text{t}}$ represents the number of false negatives at time t, ${\text{FP}}_{\text{t}}$ denotes the number of false positives at time t, ${\text{IDSW}}_{\text{t}}$ corresponds to the number of identity switches at time t.


$$\begin{array}{c} \text{IDF1=}\frac{2\times \text{IDTP}}{2\times \text{IDTP}+\text{IDFP}+\text{IDFN}} \end{array}$$ (12)

where IDTP denotes identity true positives, IDFP denotes identity false positives, IDFN denotes identity false negatives.


$$\begin{array}{c} \text{AssA=}\frac{\sum_{\text{t}}{\text{TP}}_{\text{assoc}}}{\sum_{\text{t}}({\text{TP}}_{\text{assoc}}+{\text{FP}}_{\text{assoc}}+{\text{FN}}_{\text{assoc}})} \end{array}$$ (13)

where ${\text{TP}}_{\text{assoc}}$ denotes the number of correctly matched target association pairs, ${\text{FP}}_{\text{assoc}}$ denotes the number of incorrectly matched pairs, and ${\text{FN}}_{\text{assoc}}$ denotes the number of unmatched pairs.


$$\begin{array}{c} \text{DetA=}\frac{\sum_{\text{t}}{\text{TP}}_{\text{det}}}{\sum_{\text{t}}{\text{(TP}}_{\text{det}}{\text{+FP}}_{\text{det}}{\text{+FN}}_{\text{det}}\text{)}} \end{array}$$ (14)

where ${\text{TP}}_{\text{det}}$ denotes the number of correctly detected targets, ${\text{FP}}_{\text{det}}$ denotes the number of falsely detected targets, and ${\text{FN}}_{\text{det}}$ denotes the number of undetected targets.


$$\begin{array}{c} \text{HOTA=}\sqrt{\text{DetA}\times \text{AssA}} \end{array}$$ (15)

## 4. Experimental results

### 4.1. Tracking experiments on the MST-50 dataset

To evaluate the performance of the proposed WormSORT, we compared it against several state-of-the-art  MOT methods, including DeepSORT, StrongSORT [34], ByteTrack [35], OCSORT [36] and BotSORT [37]. All experiments were conducted under identical settings.

**Fig 4:**
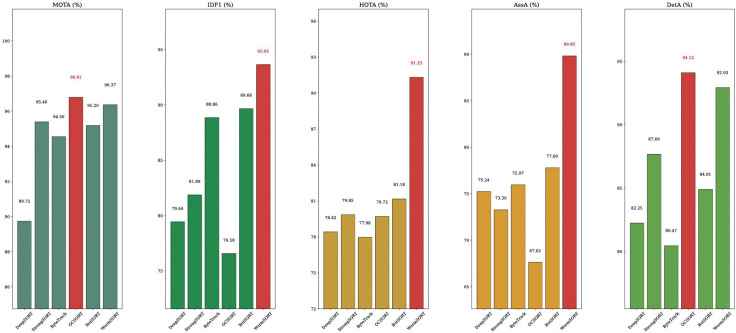
Comparison results with related methods on the MST-50 dataset.

Fig 4 presents the tracking results on the MST-50 dataset. WormSORT achieved 96.37% in MOTA, 93.65% in IDF1, 91.33% in HOTA, and 89.85% in AssA, demonstrating substantial improvements in identity consistency, association accuracy, and tracking stability. A slight decline in DetA (92.93%) was observed, likely due to the reliance on IoU-based matching and Kalman filtering, which may be less effective for nonlinear head-swinging motions of silkworms.

Compared to StrongSORT, WormSORT achieves a significant performance improvement, especially in IDF1 and AssA. This enhancement can be attributed to WormSORT’s strategy of prioritizing IoU-based matching followed by deep feature matching, combined with IoU padding and feature padding, which ensures better track association, particularly under partial occlusions and brief detection misses.

ByteTrack, which relies solely on IoU-based detection, achieved lower HOTA (77.98%) and AssA (75.97%), highlighting the limitations of detection-only association for silkworms’ motion. OCSORT, designed to mitigate occlusions using virtual trajectories, achieved high DetA (94.12%) but suffered in IDF1 (76.58%) and AssA (67.62%) due to its inability to capture nonlinear silkworm motions, indicating that virtual trajectory strategies alone are insufficient for this scenario.

BotSORT, which integrates both IoU and deep feature matching, achieved strong performance in IDF1 (89.68%) and HOTA (81.18%), but still underperformed compared to WormSORT, demonstrating the benefit of the proposed padding strategies and motion assumptions in accurately maintaining identities over time.

Overall, WormSORT achieves the best tracking performance across most evaluation metrics, effectively balancing accurate detection association and robust identity preservation, thereby demonstrating its suitability for silkworm MOT in this dataset.

**Fig 5:**
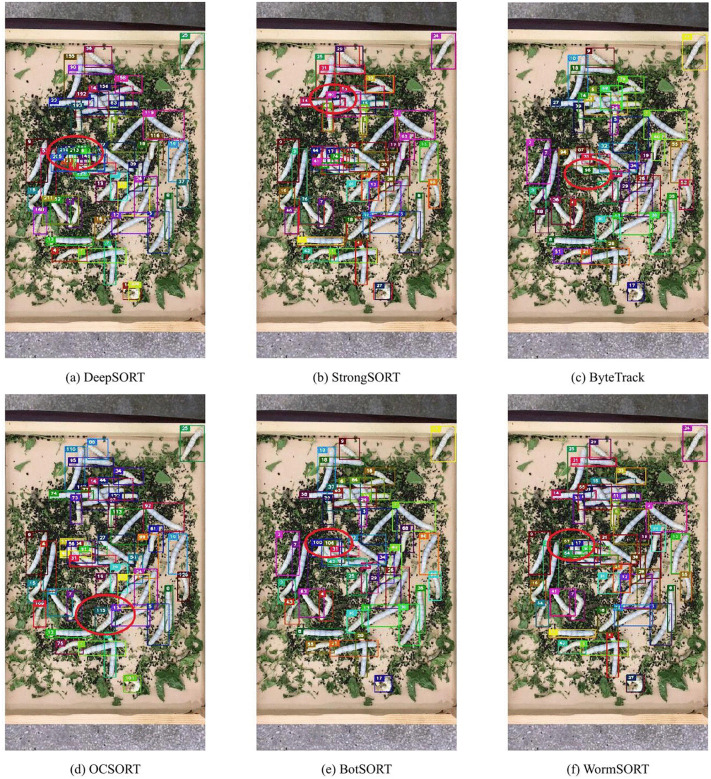
Tracking comparison of six methods at Frame 800 in MST-50 dataset.

Fig 5 presents a qualitative comparison of six methods at a late tracking stage (frame 800), highlighting identity switch behavior. The maximum assigned identity (ID) in each method is marked with red ellipses. DeepSORT reaches a maximum ID of 213, followed by StrongSORT (80), ByteTrack (96), OCSORT (115), and BoTSORT (106). In contrast, WormSORT yields a substantially lower maximum ID of 67. Although occasional short-lived detections (e.g., low-quality or transient targets) may lead to slight increases in ID count, the visualization indicates that WormSORT effectively suppresses identity switches and maintains superior tracking stability.

In summary, WormSORT demonstrates clear advantages in MOTA, IDF1, HOTA, and AssA, while maintaining competitive performance in DetA. These results collectively confirm that WormSORT achieves the best overall performance for multiple silkworm tracking.

### 4.2. Tracking experiments on the MST-100 dataset

The detector plays a critical role in multiple object tracking, especially in scenarios with complex motion and frequent occlusion such as silkworm tracking. In practice, directly applying a detector trained on general datasets often leads to suboptimal tracking performance due to domain differences. To better understand this effect, we construct task-specific training sets by sampling images from the MST-100 dataset and retrain the detector under different data scales and training strategies (Transfer learning and training from scratch). The resulting detectors are then integrated with WormSORT for tracking evaluation.

**Fig 6:**
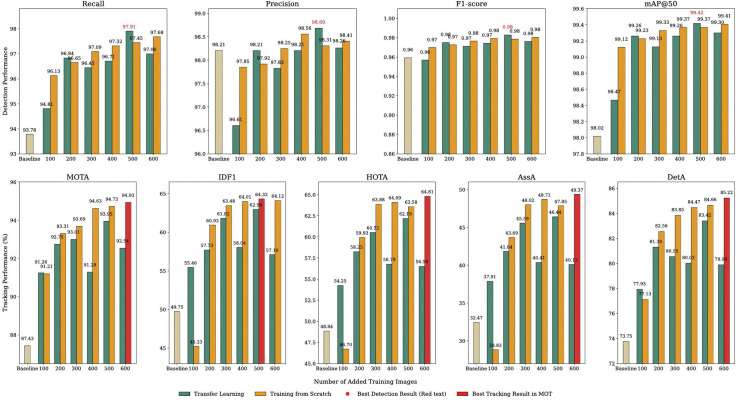
Tracking performance with different detector training strategies on the MST-100 dataset.

Fig 6 presents the tracking results of WormSORT on the MST-100 dataset with different detector configurations, demonstrating that the detector training strategy has a significant influence on tracking performance. For the baseline model, where no images from MST-100 were included in the training set, but were used entirely as the test set, the detector achieved a Recall of 93.78%, a Precision of 98.21%, an F1-score of 0.96, and an mAP@50 of 98.02%. These results are highly competitive in object detection, indicating strong detection capability. However, when directly applied to silkworms tracking, the performance is relatively limited, with IDF1 and AssA only reaching 49.75% and 32.47%, respectively.

Based on the baseline model, transfer learning not only reduces training cost but also enables the detector to quickly adapt to the distribution of silkworms in the MST-100 dataset, leading to improved detection performance. When 100 images are sampled for training, Recall increases from 93.78% to 94.81%, and mAP@50 improves from 98.02% to 98.47%, while Precision decreases from 98.21% to 96.61%. Despite this, all tracking metrics show noticeable improvements.

As the number of training images increases, the performance exhibits certain fluctuations. When the dataset size increases from 200 to 300 images, the Recall slightly decreases, while tracking performance continues to improve. A similar decrease in Recall is observed when increasing the dataset size from 500 to 600 images. These observations suggest that improvements in detection metrics do not always directly translate into better tracking performance, and the relationship between detection and tracking is not strictly monotonic.

When increasing the dataset size from 300 to 400 images, both Recall and Precision improve, but tracking performance declines. For example, IDF1 decreases from 61.82% to 58.04%, and HOTA decreases from 60.52% to 56.79%. This indicates that better detection accuracy does not necessarily guarantee improved data association quality. Among all transfer learning configurations, the detector trained with 500 images achieves the best overall performance in both detection and tracking metrics. Overall, transfer learning improves tracking performance compared to the baseline, but increasing the training data does not always lead to consistent gains, and performance fluctuations can be observed.

Training from scratch generally requires more computational resources and training time, but produces more stable trends in this task. When 100 images are added to the training set, Recall increases from 93.78% to 96.13%, while Precision decreases from 98.21% to 97.85%. Both F1-score and mAP@50 improve. However, in tracking evaluation, although MOTA and DetA increase, IDF1, HOTA, and AssA decrease significantly, indicating that the limited amount of data is insufficient for learning robust representations.

As more images are added, the detector performance improves steadily. Except for a slight decrease in Precision at 500 and 600 images, Recall shows a consistent upward trend, while F1-score and mAP@50 either improve or remain stable. In terms of tracking performance, MOTA and DetA increase steadily with the number of training images and reach their highest values at 600 images, with 94.93% and 85.22%, respectively. IDF1 reaches its peak at 500 images (64.32%) and shows a slight decrease at 600 images, which may be attributed to minor noise introduced with increased detections. For HOTA and AssA, a slight decrease is observed when increasing the dataset size from 400 to 500 images, possibly due to the reduction in Precision. Other metrics generally show an increasing trend, and both HOTA and AssA achieve their best values at 600 images, reaching 64.81% and 49.37%, respectively.

The results demonstrate that the performance of the detector has a substantial impact on tracking, including both the training data and the training strategy. Otherwise, even if competitive detection metrics such as Precision or mAP are achieved, the tracking performance may still be unsatisfactory. From the MST-100 experiments, transfer learning tends to improve detection metrics, but such improvements do not always lead to better tracking performance. In contrast, training from scratch, although more computationally expensive, generally yields better overall tracking performance. Therefore, sampling images from the target scenario and training the detector from scratch is a key step for achieving reliable tracking results in the silkworm tracking task.

Under the condition that Precision is maintained at a reasonable level, Recall plays an important role in improving multiple object tracking performance. Higher Recall provides more candidate detections, which helps maintain trajectory continuity. Missing detections, on the other hand, are more likely to cause identity switches, leading to lower IDF1 and AssA scores.

Compared to detection, improving tracking performance remains more challenging, especially in complex and long-term scenarios. This is reflected in the relatively lower values of IDF1, HOTA, and AssA. The main reason is that tracking operates under dynamic conditions, where errors accumulate over time. Missing detections or slight noise can lead to track fragmentation, and once an identity is lost or mismatched, it may trigger a cascade of tracking errors, significantly degrading reliability, even when the detector provides largely correct results.

In practical scenarios, occlusion and head movement of silkworms may further complicate data association. From a biological perspective, avoiding overly crowded conditions and excessively long tracking durations can help better capture the crawling trajectories of silkworms and ensure the reliability of subsequent analyses.

**Fig 7:**
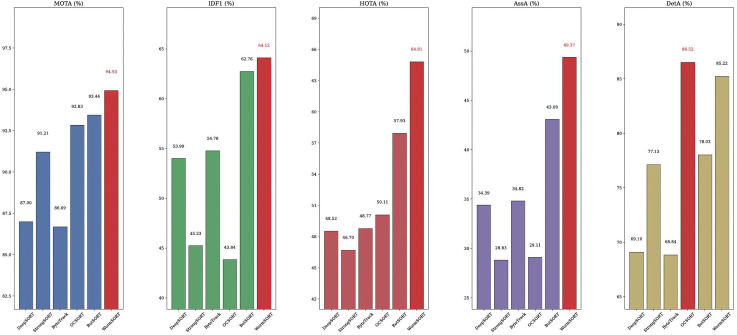
Comparison results with related methods on the MST-100 dataset.

After analyzing the impact of different detector training strategies on tracking performance, the detector that achieved the best results with WormSORT was selected for further comparison experiments. The tracking results on the MST-100 dataset are shown in Fig 7. Compared with other tracking methods, WormSORT achieves the best performance in terms of MOTA, IDF1, HOTA, and AssA, while ranking second in DetA, only slightly lower than OCSORT. These results indicate that, compared with existing methods, the data association strategy adopted in WormSORT remains effective in complex scenarios. Overall, WormSORT demonstrates good adaptability to the silkworm tracking task, although there is still room for improvement in certain metrics.

**Fig 8:**
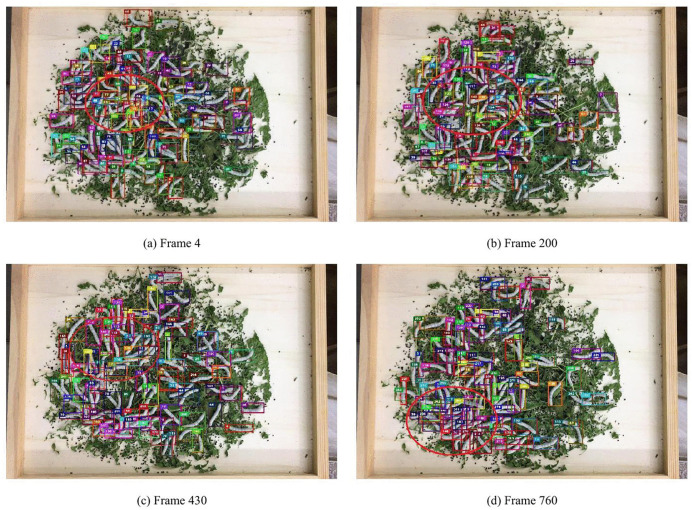
Example tracking results of WormSORT on MST-100. **(a)** Frame 4. **(b)** Frame 200. **(c)** Frame 430. **(d)** Frame 760.

Fig 8 illustrates representative tracking results of WormSORT on the MST-100 dataset. Red ellipses highlight densely populated regions where identity switches are more likely to occur. At Frame 4, the IDs in this region range approximately from 60 to 90. By Frame 200, the ID range increases to 140–150, and further rises to nearly 200 by Frame 430. At Frame 760, due to silkworm movement, the dense region shifts to the lower-left area, where the ID values increase to approximately 260.

**Fig 9:**
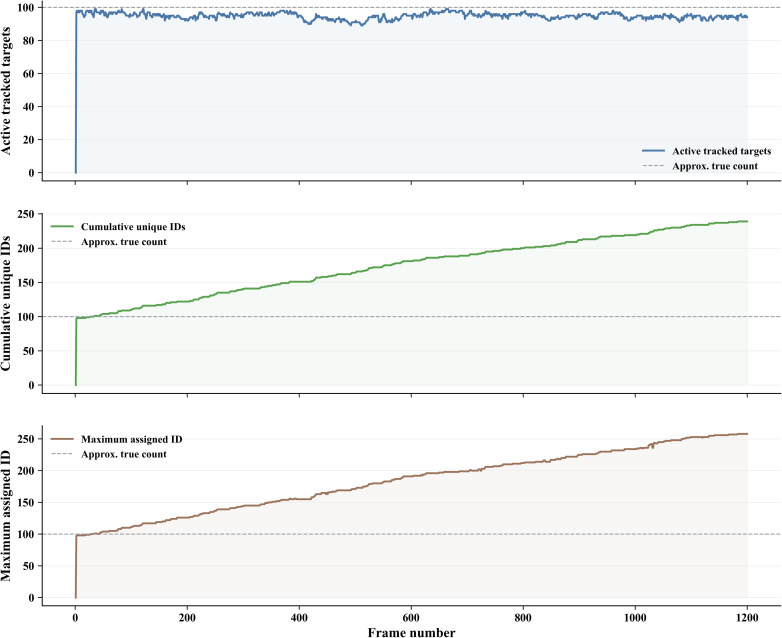
Temporal evolution of tracking behavior of WormSORT on the MST-100 sequence.

Fig 9 shows that the number of active tracked targets remains close to the true population size throughout MST-100. whereas the cumulative number of unique IDs increased steadily from approximately 100 to about 240, and the maximum assigned ID exceeded 250 at the end of the sequence. This indicates that the long-sequence degradation of WormSORT is mainly caused by cumulative identity fragmentation and ID switches, rather than substantial target loss. Combined with the qualitative results in Fig 8, these failures are primarily associated with prolonged overlap in densely populated regions, and are further aggravated by abrupt head swings that are not well captured by the Kalman filter.

Notably, WormSORT and other compared methods exhibit relatively low IDF1 and AssA scores on MST-100, indicating that heavy spatial crowding significantly degrades tracking performance. In densely distributed regions, frequent positional overlap leads to high IoU between targets, while appearance features become insufficiently discriminative. This inevitably results in identity switches. These observations suggest that severe crowding is the primary factor limiting tracking accuracy. Therefore, when applying MOT to silkworm trajectory analysis, avoiding excessively dense scenarios is essential for achieving reliable tracking performance.

### 4.3 Ablation analysis

To further validate the contribution of each component in WormSORT, ablation experiments were conducted on the MST-50 and MST-100 datasets. Method (a) first performs IoU-based matching followed by appearance-feature matching. Based on this baseline, IoU-padding was introduced in Method (b), feature-padding in Method (c), and both strategies were combined in Method (d), which corresponds to the full WormSORT framework.

As shown in Table 3, the baseline method (a) already achieved strong performance on MST-50, with a MOTA of 96.54%, IDF1 of 92.97%, and HOTA of 87.01%, indicating that the IoU-first matching strategy is effective for silkworm MOT. This result is consistent with the motion characteristics of silkworms, whose trajectories are relatively smooth and do not exhibit abrupt displacement between adjacent frames. Under such conditions, IoU provides reliable association cues, whereas appearance features are less discriminative when targets are densely distributed or partially overlapping.

**Table 3:** Ablation results of WormSORT.

Method	MOTA (%)		IDF1 (%)		HOTA (%)		AssA (%)		DetA (%)	
	MST-50	MST-100	MST-50	MST-100	MST-50	MST-100	MST-50	MST-100	MST-50	MST-100
(a)	**96.54**	91.71	92.97	56.59	87.01	55.66	86.78	40.91	87.62	76.65
(b)	96.52	92.00	93.06	63.15	87.15	63.05	87.12	48.14	87.55	82.73
(c)	95.03	89.07	**93.68**	62.16	86.35	61.31	87.23	46.97	85.81	80.16
(d)	96.37	**94.93**	93.65	**64.12**	**91.33**	**64.81**	**89.85**	**49.37**	**92.93**	**85.22**

Introducing IoU-padding in Method (b) led to consistent improvements, particularly on the more challenging MST-100 dataset. Compared with Method (a), MOTA increased from 91.71% to 92.00%, IDF1 from 56.59% to 63.15%, HOTA from 55.66% to 63.05%, AssA from 40.91% to 48.14%, and DetA from 76.65% to 82.73%. On MST-50, the gains were smaller but still observable in IDF1, HOTA, and AssA. These results indicate that IoU-padding effectively preserves high-confidence unmatched trajectories and detections for subsequent association, thereby reducing missed matches and improving both association quality and detection reliability.

By contrast, feature-padding alone in Method (c) produced mixed results. On MST-50, IDF1 increased slightly from 92.97% to 93.68%, but MOTA and HOTA decreased to 95.03% and 86.35%, respectively. A similar trend was observed on MST-100, where Method (c) improved IDF1 and AssA relative to the baseline but remained inferior to Method (b) across all metrics. This suggests that although feature-padding can provide supplementary cues for identity preservation, its contribution is limited when appearance features are insufficiently discriminative, especially in crowded scenes where severe overlap occurs among silkworm targets.

When IoU-padding and feature-padding were jointly applied in Method (d), the best overall performance was achieved on both datasets. On MST-50, Method (d) yielded 96.37% MOTA, 93.65% IDF1, 91.33% HOTA, 89.85% AssA, and 92.93% DetA. On MST-100, the corresponding values reached 94.93%, 64.12%, 64.81%, 49.37%, and 85.22%, respectively, all of which were the best among the four methods. Notably, the combined strategy produced the largest gains in HOTA, AssA, and DetA, indicating that the two padding mechanisms are complementary: IoU-padding primarily strengthens motion- and location-based association, while feature-padding further improves identity continuity in ambiguous cases.

Overall, the ablation results demonstrate that IoU-first matching is well suited to silkworm tracking, and that the proposed padding mechanisms, particularly when combined, substantially improve tracking robustness and association accuracy. These findings confirm the effectiveness of the full WormSORT design.

Table 4 reports the impact of the IoU-padding threshold δ on tracking performance. Overall, δ = 5 provides the best trade-off across both datasets, achieving the highest IDF1, HOTA, and AssA on MST-100, while maintaining stable performance on MST-50. When δ is too small (δ = 3), the candidate set becomes overly restrictive, leading to missed associations; conversely, a larger threshold (δ = 7) introduces more ambiguous candidates, slightly degrading association quality. These results indicate that a moderate motion constraint is essential for balancing recall and reliability in IoU-based candidate selection.

**Table 4:** Ablation results of different δ values.

δ	MOTA		IDF1		HOTA		AssA		DetA	
	MST-50	MST-100	MST-50	MST-100	MST-50	MST-100	MST-50	MST-100	MST-50	MST-100
3	95.28	91.96	**93.32**	63.09	**90.72**	62.95	**89.61**	47.98	91.20	82.72
5	**96.52**	**92.00**	93.06	**63.15**	87.15	**63.05**	87.12	**48.14**	87.55	82.73
7	95.50	91.57	93.23	62.96	90.55	62.63	89.10	47.71	**92.10**	**82.91**

Table 5 presents the ablation results for different feature-padding thresholds ε. As ε increases, stricter cosine similarity constraints improve identity discrimination, with ε = 0.8 achieving the highest IDF1 on both datasets. However, this also leads to reduced MOTA, HOTA, and DetA, as fewer candidates are retained for matching. In contrast, lower thresholds (ε = 0.4, 0.6) allow more flexible matching and yield better overall detection–association coverage. These findings suggest that feature-padding primarily refines identity consistency, but requires a careful balance to avoid over-restricting valid associations.

**Table 5:** Ablation results of different ε values.

ε	MOTA		IDF1		HOTA		AssA		DetA	
	MST-50	MST-100	MST-50	MST-100	MST-50	MST-100	MST-50	MST-100	MST-50	MST-100
0.4	94.72	**90.10**	92.96	62.05	**90.09**	61.37	**88.89**	46.50	**91.40**	**81.14**
0.6	94.50	90.01	92.86	62.14	89.93	**61.59**	88.77	46.90	91.18	81.01
0.8	**95.03**	89.07	**93.68**	**62.16**	86.35	61.31	87.23	**46.97**	85.81	80.16

## 5. Discussion

In this study, we applied artificial intelligence to silkworm breeding and proposed a MOT method for silkworms. Unlike existing deep learning-based silkworm recognition methods, such as individual detection [38], image classification [39], or identity re-identification [40], MOT is a more complex and versatile technique. It integrates object detection and identity re-identification to match the same individual across consecutive images. This enables researchers to track silkworms’ positional changes over time, providing valuable insights into their behavior and interaction mechanisms. Despite challenges such as high individual similarity, irregular movement patterns, and head swings, the results show that the tracking-by-detection (TBD) method effectively associates silkworm data. Moreover, compared to static image-based silkworm detection [20], this study offers a dynamic individual counting method that reduces misdetections and missed detections due to temporary occlusions.

Unlike tracking vehicles or pedestrians, where motion direction and speed are relatively consistent (e.g., vehicles do not abruptly change direction), silkworms exhibit nonlinear head swings that disrupt the filter. Additionally, after head movement, the depth feature vectors of the same silkworm can change significantly. As a result, tracking using DeepSORT or StrongSORT under the same detector performs poorly. This highlights the importance of position-based matching, with deep feature matching used as a supplementary strategy in silkworm tracking. Position-based matching mitigates the filter’s vulnerability to nonlinear predictions [41, 42], and the proposed padding mechanism increases the system’s tolerance for data management. This is particularly important in dense environments, where detectors may fail to detect individuals, and feature matching excels at recovering these individuals.

Despite outperforming other methods on silkworm tracking datasets, WormSORT has limitations. Its reliance on IoU-based matching makes it prone to losing individual silkworms [43]. Some silkworms remain untracked for extended periods, and performance degrades over longer tracking sequences. These issues may arise from declining detector performance or overlapping individuals.

Future improvements could focus on enhancing the model’s adaptability to nonlinear silkworm motion—for instance, by optimizing the filter based on motion patterns—and better balancing IoU- and feature-based matching to fully exploit their respective strengths [44]. Incorporating more advanced data association strategies from the broader MOT field, especially those designed to handle occlusions, could further improve robustness in challenging tracking scenarios.

## 6. Conclusion

In this study, we introduced WormSORT, a tracking-by-detection (TBD)-based multiple object tracking (MOT) method for silkworm tracking in breeding environments. Based on the observation that silkworm positions usually change smoothly between adjacent frames, WormSORT prioritizes IoU-based matching, followed by deep feature matching for unmatched targets, thereby achieving efficient and reliable data association. To further improve robustness under complex conditions, IoU-padding and feature-padding mechanisms were incorporated to retain high-confidence tracks and detections in the candidate set for subsequent association.

Experimental results on the proposed datasets demonstrate the effectiveness of WormSORT for silkworm MOT. The main conclusions are as follows:

(1) WormSORT provides an effective framework for long-duration silkworm tracking. By prioritizing IoU-based matching and using deep feature matching as a complementary strategy, the method achieves reliable target association. The proposed IoU-padding and feature-padding mechanisms further improve tracking robustness by preserving high-quality unmatched candidates for subsequent matching.
(2) Detector quality plays a critical role in silkworm MOT. Although high detection metrics can be obtained in the object detection task, they do not necessarily translate into satisfactory tracking performance. The results show that sampling images from the target tracking scenario and training the detector accordingly is essential for achieving stable and reliable tracking.
(3) WormSORT achieves the best overall performance on both MST-50 and MST-100 among the compared methods. However, performance remains limited in highly crowded scenes, where dense spatial overlap is the main cause of identity fragmentation and ID switches. This indicates that severe crowding remains the primary challenge for accurate silkworm MOT.

Overall, WormSORT provides an efficient, non-invasive, and dynamic approach for silkworm tracking and individual counting in breeding environments. It also offers a useful technical basis for future studies on silkworm behavior analysis and the identification of abnormal or diseased individuals from trajectory patterns.
